# A Sub-ppm Acetone Gas Sensor for Diabetes Detection Using 10 nm Thick Ultrathin InN FETs

**DOI:** 10.3390/s120607157

**Published:** 2012-05-29

**Authors:** Kun-Wei Kao, Ming-Che Hsu, Yuh-Hwa Chang, Shangjr Gwo, J. Andrew Yeh

**Affiliations:** 1 Institute of NanoEngineering and MicroSystems, National Tsing Hua University, Hsinchu 30013, Taiwan; E-Mails: s9835812@m98.nthu.edu.tw (K.-W.K.); d929205@oz.nthu.edu.tw (Y.-H.C.); 2 Institute of Electronics Engineering, National Tsing Hua University, Hsinchu 30013, Taiwan; E-Mail: s9863570@m98.nthu.edu.tw; 3 Department of Physics, National Tsing Hua University, Hsinchu 30013, Taiwan; E-Mail: gwo@phys.nthu.edu.tw

**Keywords:** indium nitride (InN), acetone, gas sensor, diabetic, platinum (Pt)

## Abstract

An indium nitride (InN) gas sensor of 10 nm in thickness has achieved detection limit of 0.4 ppm acetone. The sensor has a size of 1 mm by 2.5 mm, while its sensing area is 0.25 mm by 2 mm. Detection of such a low acetone concentration in exhaled breath could enable early diagnosis of diabetes for portable physiological applications. The ultrathin InN epilayer extensively enhances sensing sensitivity due to its strong electron accumulation on roughly 5–10 nm deep layers from the surface. Platinum as catalyst can increase output current signals by 2.5-fold (94 *vs.* 37.5 μA) as well as reduce response time by 8.4-fold (150 *vs.* 1,260 s) in comparison with bare InN. More, the effect of 3% oxygen consumption due to breath inhalation and exhalation on 2.4 ppm acetone gas detection was investigated, indicating that such an acetone concentration can be analyzed in air.

## 1. Introduction

Over two hundred kinds of volatile organic compounds (VOCs) are found in human breath while the concentrations of such VOCs are usually measured to be at sub-ppm level or even lower for healthy human beings [1]. Abnormal concentrations of the breath VOCs are reported to correlate with unhealthy/injurious body/organ conditions; for instance, acetone gas for diabetes [2], trimethylamine for uremic patients [3] and ammonia gas for renal disease [4]. Hence, VOCs can potentially be used as disease-specific biomarkers for non-invasive early detection or monitoring from breath. Acetone can be produced via the fatty acid oxidation in diabetes and ketoacidosis lack of insulin [5]. Excessive acetone circulating in the blood system is excreted from the lungs. Higher acetone concentration ranges from 1.7 ppm to 3.7 ppm could be detected in breath for those who are diabetic, while the breath of healthy human typically contains less than 0.8 ppm [6]. Gas sensors with sub-ppm acetone detection capacity play an important role in the development of non-invasive monitors or early diagnosis of potential diabetic patients.

Sub-ppm traces of VOCs can be analyzed easily using table-top equipments, such as Gas Chromatography-Mass Spectrometry (GC-MS) and Proton Transfer Reaction-Mass Spectrometry (PTR-MS); however, such equipment does not meet the requirements of clinical or at-home applications, including portability, small form factor, cost-effective performance, real-time analysis and so forth. Alternative approaches proposed to detect gas traces in low concentrations include electrochemical sensors [7], surface acoustic wave [8], quartz crystal microbalance [9–11], and semiconductor gas sensors. Among them, gas sensors based on semiconductor materials, viewed as electronic devices, can be further capable of integration with electronic circuitries. The semiconductor materials generally used are Group V (e.g., (porous) silicon [12], carbon (nanotubes) [13]), Group II-VI (*i.e.*, metal oxides [14–17]) and Group III-V (*i.e.*, metal nitrides [18]). In comparison, Group III-nitrides display the unique properties of high electron concentration near the surface, long-term chemical stability, bio-compatibility and high sensitivity [19–23], offering great potential in detecting chemical species and biological molecules [24–29].

Table 1 shows the comparison of various gateless (*i.e.*, unbiased) acetone gas sensors that are made based on metal oxides or nitrides. Metal oxide materials, such as Fe_2_O_3_ [14], In_2_O_3_ [15], WO_3_ [16], ZnO [17,30,31], LaFeO_3_ [32] and TiO_2_ [33] were demonstrated, but the majority cannot achieve the sub-ppm acetone detection with high sensitivity and high linearity. Note that the response time varies to different ambient environments (e.g., N_2_ and air) since air is composed of oxygen, which also reacts to the sensors. The response time, defined by change of from 10% to 90%, also depends on the acetone concentration and on the operation temperature. The higher the acetone concentration the sensor is exposed to, the shorter the response time.

In this study, acetone gas sensors developed on ultrathin (∼10 nm) indium nitride (InN) epilayers are proposed [25]. Sensitivity is expected to be much better due to the natural electronic characteristics of as-grown InN films, including a narrow band gap, excellent electron transport characteristics (mobility > 1,000 cm^2^·V·s), a background high electron density (typically in excess of 1 × 10^18^ cm^−3^), and the unusual phenomenon of strong surface electron (charge) accumulation (1.57 × 10^13^ cm^−2^) for the InN films grown on AlN film on a buffer layer [34,35].

The surface electron accumulation along with the high mobility induces a large current change upon exposure to charge varying environments. The excessive surface electrons are typically manifested by a high sheet density on the region within 5 nm away from the surface of InN; thus, the film used in this study is designed to be about 10 nm. The surface charge accumulation layer forms a natural two-dimensional electron gas (2DEG) on the surface, beneficial to chemical detection. More, InN is a chemically stable semiconductor with great resistance to either strong acid or strong base [28].

## 2. Experimental Section

The responses of gateless InN sensors to acetone gas were investigated for acetone concentrations of 0.4 ppm to 20 ppm in synthetic air using conductivity measurements. A thin catalytic Pt film of 10 nm in thickness was further characterized to analyze its effect on acetone sensing. Experiments on sensitivity, detection limit, response time, recovery rate, and long-term stability are conducted.

### 2.1. Performance Parameters of InN Gas Sensor

For gas sensors, three main parameters are taken into account, including sensitivity, limit of detection and response (rise/fall) times. The sensitivity is usually defined as the current variation ratio of the measured gas concentration. For example, if the background gas is air, the gas sensitivity is defined as follows:

(1) $$\text{Sensitivity}(\text{S})\equiv \frac{{\text{I}}_{\text{gas}}-{\text{I}}_{\text{air}}}{{\text{I}}_{\text{air}}}$$

where I_gas_ and I_air_ are the current values when the gas sensor is exposed to acetone in air, respectively.

Limit of detection is defined as the minimum value of analyte concentration that can produce a sufficiently different signal from the signals of a blank sample. In this study, the value of detection limit is obtained when the sensor removed the adsorbed water molecules from the sensor surface. The limit of sensor detection is defined as the value of sensor sensitivity that is greater than three times the standard deviation of the noise signal. In this paper, the maximum standard deviation of the noise signal is 0.354 μA. In terms of the response and recovery times, they are defined as the elapsed times of the relative current change from 10% to 90% and from 90% to 10%, respectively.

### 2.2. Fabrication of InN Gas Sensor and Experimental Setup

**Figure 1.**
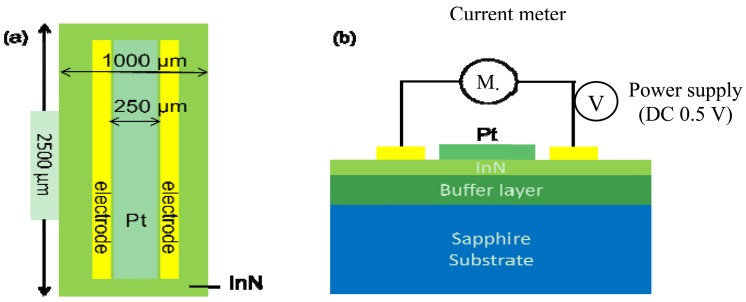
Schematic structure of a Pt-InN acetone sensor Pt-InN acetone sensor (**a**) Top view; (**b**) Side view and current measurement connections.

The gas sensors made on In-polar InN epilayers were comprised of a pair electrodes of Au/Al/Ti (50 nm/200 nm/50 nm) composite for current-voltage (I–V) measurement and a sensing window (250 μm in width × 2,000 μm in length) exposed to the ambient gas environment. The two types of InN acetone gas sensors fabricated are denoted as bare InN and Pt-InN, respectively. The Pt-InN one was deposited with a 10 nm thick Pt film on the sensing window to investigate the effect of catalyst on acetone gas detection (see Figure 1) The In-polar InN epilayer was grown on c-plane sapphire substrates with an AlN (0001) buffer layer by using a plasma-assisted molecular beam epitaxy (PAMBE) system. All metal films used in this study were deposited by an electron-beam evaporation system. After a lithography process, to define the sensing device size the sensing device was attached to the heater using a thermally conductive silicon adhesive (Dow Corning SE4485, 2.5 W/m·K) that assists with the heat conduction. Finally, the sensor is fixed and isolated from other metal contact with a UV curing adhesive.

The experiments with the sensors were conducted in a stainless steel chamber with respect to various acetone concentration ranging from 0.4 ppm to 20 ppm at temperatures of 150, 175 and 200 °C. Note that the acetone concentration in the normal breath is less than 0.8 ppm while for type II diabetes it exceeds 1.8 ppm. The acetone concentrations in the chamber were adjusted by varying the flow rate ratio of two gas channels (acetone and synthetic air) through mass flow controllers (MFCs, MKS Type 1179A), manipulated by a real-time monitor computer in this experimental system. The synthetic air was pre-mixed with 21% oxygen gas and 79% nitrogen gas. The sensors were biased at 0.5 V by a dc power supply (Agilent 6645A) with an internal GPIB interface and programming. A serpentine aluminum microheater was attached onto the backside of a sensor. The temperature read via a K-type thermal couple was applied as real-time feedback signal to reach variation control of within ±0.1 °C using a FLUKE 2640A NetDAQ with 5½ digits of resolution. The signals were transmitted via wire bonds across the sensor pads and the PCB board, and on to the measurement instruments. The current flow through the two electrodes was recorded by a multimeter with 7½-digit resolution (Keithley model 2001).

## 3. Results and Discussion

**Figure 2.**
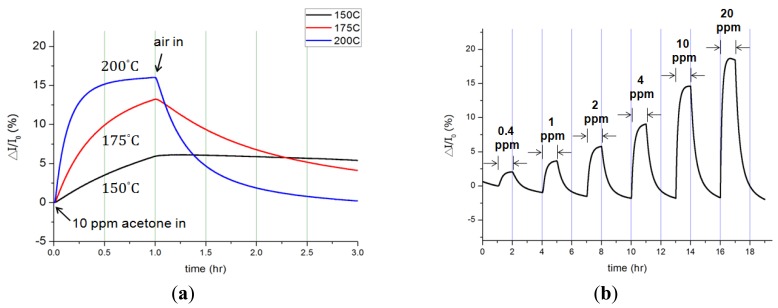
Bare InN acetone sensor (**a**) Response at various temperatures of 150 °C, 175 °C and 200 °C in 10 ppm acetone/air mixture ambiance; (**b**) Response to various acetone concentrations of 0.4 ppm–20 ppm at the temperature of 200 °C.

The influences of acetone concentration, temperature and Pt catalyst on InN gas sensors were characterized and analyzed. Figure 2 shows the dynamic responses of bare InN acetone gas sensors: (a) to various temperatures from 150 °C to 200 °C upon exposure to 10 ppm acetone in synthetic air and (b) to various acetone concentrations ranging from 0.4 ppm to 20 ppm at 200 °C. The measured current variation (Δ*I*) and the calculated current variation ratio (Δ*I/I*_0_) were recorded under a bias of 0.5 V across two electrodes. Figure 2(a) implies that as the temperature increases, both the response and recovery times shortened along with enlarged current signals. At 150 °C, the bare-InN sensor reacted to the gas composition, but it barely returned to its original state after 2 h. As the sensor temperature increased to 175 °C, the current variation ratio also increased to 13.2%. The maximum current variation of 37.5 μA was observed at 200 °C, corresponding to a variation ratio of 16%.

**Figure 3.**
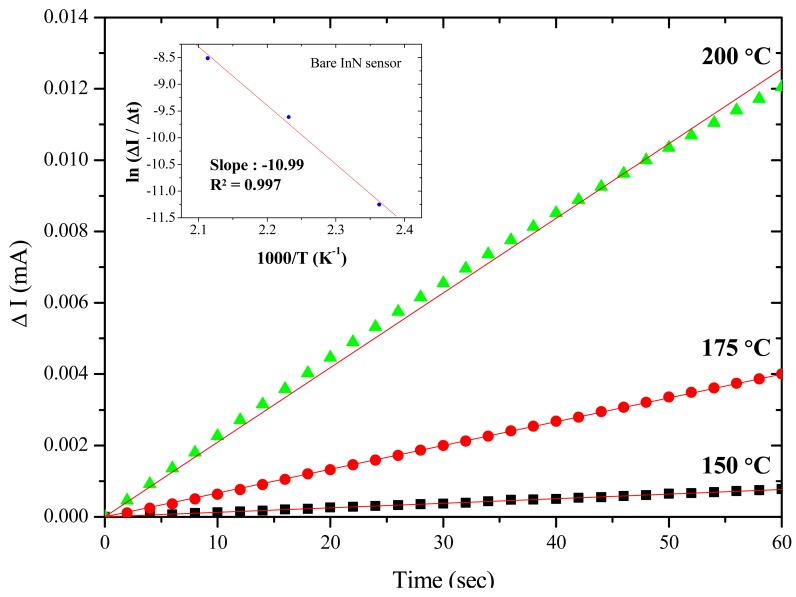
Transient response of current variation (ΔI) for the Bare InN sensor in the first 60 s upon exposure to 10 ppm acetone/air at different temperatures from 150 to 200 °C.

Further, the activation energy of acetone gas reacting to the bare InN sensor is derived by extracting the slope on the Arrhenius plot of the current variation rate (Δ*I*/Δ*t*) *vs.* the inverse of temperature (1/T). The activation energy is determined based on the highest current variation rate in the first 60 s upon exposure to 10 ppm acetone/synthetic air at the temperatures of 150, 175 and 200 °C. The inset in Figure 3 shows the Arrhenius plot, yielding an activation energy of 21.9 kcal/mol (*i.e.*, 0.95 eV). A similar result of 0.87 eV also can be found in other studies [36]. The current variation, current variation ratio, and response/recovery times at different temperatures are summarized in Table 2. The temperature increase can accelerate gas sensing reactions, but should not be more than the maximum operating temperature (200 °C) to prevent the degradation of the InN film.

The current variation (Δ*I*) of a bare InN acetone sensor under exposure to various acetone concentrations from 0.4 ppm to 20 ppm in synthetic air at 200 °C is depicted in Figure 2(b). The experiments were repeatedly performed while the acetone gas was turned on for an hour and off (*i.e.*, in pure synthetic air) for another two hours at various acetone concentrations. The current went up as acetone concentration increased; such current increment was induced by the reduction of pre-adsorbed oxygen atoms [37] The originally adsorbed oxygen atoms on the InN surface tend to become negatively charged (*i.e.*, acceptors) due to the charge transfer from the surface conduction band to the adsorbed oxygen. The charge transfer results in the formation of surface depletion layers, thus causing a decrease of the surface conductivity of the InN film. When acetone (CH_3_COCH_3_) molecules are introduced to the sensing system, the hydrogen atoms on dissociated acetone molecules react with pre-adsorbed oxygen atoms, reducing the effects of surface depletion. As a result, both the influences of reduction of pre-adsorbed oxygen atoms and adsorption of acetone molecules contribute more electrons, increasing the surface conductivity of the InN. The dynamic responses of the bare InN sensor indeed demonstrate the great ability to sense acetone gas at the sub-ppm level.

**Figure 4.**
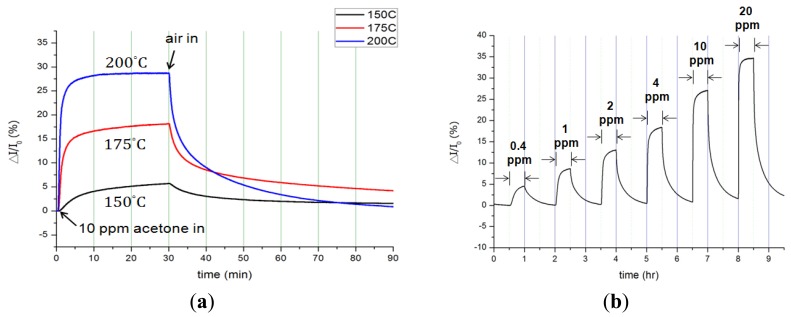
Pt-InN acetone sensor (**a**) Response at various temperatures from 150 °C to 200 °C in 10 ppm Acetone/air mixture ambiance; (**b**) Response of various acetone concentration (0.4 ppm–20 ppm), operating temperature at 200 °C.

Distinct to a bare InN sensor, the InN sensor coated with Pt catalyst (Pt-InN) was also characterized (see Figure 4). The responses of current variation ratio of a Pt-InN acetone sensor upon exposure to 10 ppm acetone/air at various temperatures from 150 °C to 200 °C are shown in Figure 4(a). In comparison with the bare InN sensor, the Pt-coated sensor yields a higher sensitivity and faster response/recovery rates at the same temperature due to the catalytic effect of the Pt. At 150 °C, the sensor exhibits a current variation ratio of 5.7% upon exposure to 10 ppm acetone/air. As the operating temperature rises to 200 °C, the current variation ratio increases to 28.7% and the response/recovery times shorten to 150/2,000 s. The response and recovery times significantly decrease by 7-fold with increasing temperature from 150 °C to 200 °C. The enhanced performance results from the adsorption of acetone molecules and dissociated hydrogen atoms at the outer surface of the catalytic metal (Pt) film. At the surface of the Pt film, a portion of hydrogen atoms dissociated from acetone molecules reacts with other hydrogen atoms or pre-adsorbed oxygen atoms to produce hydrogen or water molecules, respectively, leaving the Pt surface [35]. On the other hand, some dissociated hydrogen atoms diffuse rapidly through the catalytic Pt film, and then are trapped at the Pt/semiconductor interfaces. The trapped hydrogen atoms induce an interface dipole layer, affecting the sensor Pt-InN. The introduction of Pt-catalyst enhances the current variation by 2.5 times under the 10 ppm acetone/air exposure at 200 °C compared to the bare InN sensor. The current variation, current variation ratio, and response/recovery times at various temperatures from 150 °C to 200 °C are summarized in Table 3.

**Figure 5.**
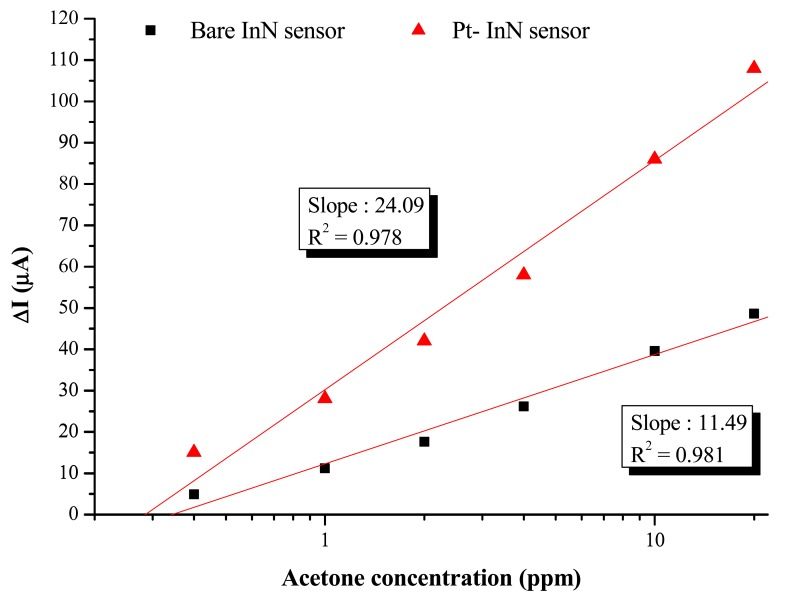
Comparison of current variation of Pt-coated InN and Bare InN.

The bare InN sensor reveals an obvious current variation response to acetone at a maximum temperature at 200 °C. The maximum response of current variation under a 10 ppm acetone exposure at 200 °C is 37.5 μA, corresponding to a variation ratio of 16% and a response time of 1,260 s. On the other hand, the Pt-InN acetone sensor exhibits a much higher response to acetone even at the same temperature. At 150 °C, the current variation ratio of 5.7% is observed while the sensor is exposed to 10 ppm acetone/air. As the temperature is elevated to 200 °C, the sensor shows a much higher current variation ratio of 28.7% and shorter response/recovery times of 150/2,000 seconds in the same gas exposure environment. Compared to a bare InN acetone sensor tested under the same conditions, the InN acetone sensor with Pt catalytic layer remarkably enhances the current variation by ∼2.5 times and significantly reduces the response/recovery time by 1,110/1,740 s (*i.e.*, 740%/87%). The comparison of current variation with Pt-coated InN and bare InN sensors is shown in Figure 5. The plot of current variation (ΔI) *vs.* log of acetone concentration reveals linear relationships of Pt-InN (R^2^ = 0.978) and bare InN (R^2^ = 0.981) sensors with slopes of 24.1 and 11.5, respectively. The slope of Pt-InN sensor substantially increases by ∼117% in this work.

Humidity and many gas in human breath was reported to affect the measurement of gas sensors [38,39]; commercial solutions are available to minimize humidity effects [40]. In this study, the sensor operates at a temperature of 200 °C, limiting the existence of humidity.

**Figure 6.**
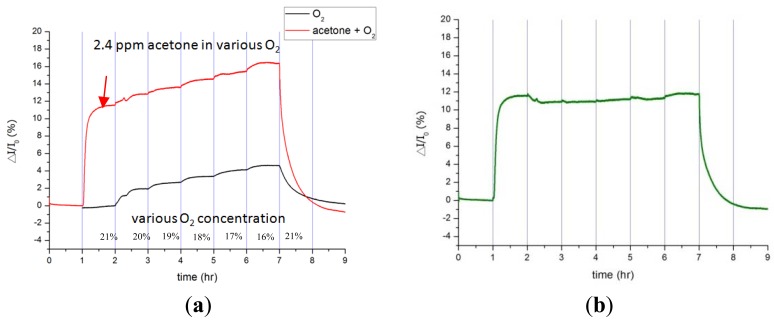
Effect of oxygen on acetone detection using the Pt-InN sensor under various oxygen concentrations of 21% to 16% (**a**) pure oxygen (red line) and oxygen with 2.4 ppm acetone (black line); (**b**) the sensor exposed to constant 2.4 ppm acetone.

Oxygen gas, which constitutes roughly 21% of the volume of air, is second to inert nitrogen gas in terms of volume. Human beings consume about 3% of oxygen gas per breath; thus, the study analyzes the influence of the oxygen volume changing from 21% to 16% in pure nitrogen. The current variation of the Pt-InN sensor increases by 10.7% to 11.9% as exposed to 2.4 ppm acetone gas during a period of six hours while that changes by 3% for 3% oxygen reduction and by 4.6% for 5% oxygen reduction (see Figure 6). Note that 2.4 ppm acetone falls in the breath concentration of reported diabetic patients. The 3% oxygen reduction between exhalation and inhalation weighs about a quarter of current response to 2.4 ppm acetone gas. The experimental results indicate that acetone gas concentration can be precisely estimated down to ppm resolution in the air environment.

The Pt-InN sensors demonstrate better gas sensing abilities, such as faster response/recovery time (150/2,000 s), higher sensitivity (28.7%) and lower limit of acetone detection (0.4 ppm) at 200 °C. From previous studies, a resistance variation ratio of ∼10.1% was observed for a sol-gel derived ZnO thin films sensor upon exposure to 1,000 ppm acetone at 200 °C [27]. A Sr-doped nanostructured LaFeO_3_ semiconductor sensor has 0.7% resistance variation ratio as exposed to 500 ppm acetone at the higher operating temperature (275 °C) [28]. An acetone sensor employing electrochemically doped Ni in ZnO nanorods with enhanced ultraviolet (UV) activation has a resistance variation ratio of 1.61% under 100 ppm of acetone gas at room temperature [29]. Compared with other sensing mechanisms of semiconductor for acetone breath analysis, the Pt-InN acetone sensor proposed in this work possesses the advantages of a rapid response, simple structure and low detection limit, and shows great potential for precise monitoring of diabetes in hospital and future applications on clinical diagnosis.

## 4. Conclusions

High-sensitivity acetone detection in air using an ultrathin InN epilayer with a Pt catalyst has been demonstrated. At the operating temperature of 200 °C, a sub-ppm (0.4 ppm) order of acetone concentrations can be detected, and the Pt-InN acetone sensor exhibits a high current response with a sensitivity of ∼28.7% at 20 ppm. The response is much higher than bare InN acetone sensors operated in a normally-on mode with an unbiased gate under the similar gas exposure conditions. The higher response of acetone detection for the Pt-coated InN sensors is associated with the incorporation of the catalytically active hydrogen atoms into the near-surface region of InN, which act as donors and thus enhance the surface conduction current. The Pt catalytic layer remarkably enhances the current variation by 2.5 times in comparison with the bare InN sensor under the same conditions. Therefore, the Pt–InN sensor with simple structure and easy fabrication (two-mask process) is a promising device for the realization of non-invasive acetone sensing with high sensitivity and low detection limit (sub-ppm level) to detect acetone in exhaled breath as a marker for diabetes.

## Figures and Tables

**Table 1.** List of acetone gas sensors.

**Material**	**Principle of Operation Device Type**	**Sensitivity (% per conc. Decade Change)**	**Lowest Concentration Detected (ppm)**	**Response Time**	**Operation Temperature**

In_2_O_3_ [15]	Resistance (voltage) change Nanowire	0.6 (-)	25	∼10 s * (in N_2_)	400 °C
WO_3_ [16]	Resistance (voltage) change Nanoparticle	1.5 (1.76) *	0.2	∼3.5 m * (-)	400 °C
ZnO [30]	Resistance (voltage) change Thin Film	5.71 (-)	100	30 s (-)	200 °C
ZnO + Ni +UV light [31]	Resistance (voltage) change Nanorods	1.61 (-)	100	5 min (in air)	Room temperature
LaFeO_3_ [32]	Resistance (voltage) change Thin Film	0.7 (-)	500	33 s (in air)	275 °C
TiO_2_ [33]	Resistance (voltage) change Thin Film	<4 * (-)	1 ppm	∼10 s * (in air)	500 °C
GaN [18]	Resistance(voltage) change Thin Films	∼23 * (-)	500 ppm	10 s for 1,000 ppm (in air)	350 °C
InN (this study)	Resistance (voltage) change Thin Films	28.7 (24.1)	0.4 ppm	150 s for 10 ppm (in air)	200 °C

Note: 1. The dash “-” symbol indicates no data found in the referred paper; 2. The asterisk symbol “*” indicates data estimated from the graph in the paper.

**Table 2.** Current variation (Δ*I*), current variation ratio (Δ*I/I_0_*), and response/recovery times for a bare In-polar InN sensor under exposure to 10 ppm acetone/air at various temperatures.

**Temperature**	**150 °C**	**175 °C**	**200 °C**
ΔI/I_0_ (%)	5.9	13.2	16
ΔI (μA)	16.1	32.5	37.5
Response time	-	2,480 s	1,260 s
Recovery time	-	-	3,740 s

Note: the dash symbol “-” implies a time longer than two hours.

**Table 3.** Current variation (Δ*I*), current variation ratio (Δ*I/I_0_*), and response/recovery times for a Pt-InN acetone sensor under exposure to 10 ppm acetone/air at various temperatures.

**Temperature**	**150 °C**	**175 °C**	**200 °C**
ΔI/I_0_ (%)	5.7	18.2	28.7
ΔI (μA)	21	60	94
Response time	1,090 s	458 s	150 s
Recovery time	-	-	2,000 s

Note: the dash symbol “-” implies a time longer than one hour.

## References

[1] Manolis A. (1983). The diagnostic potential of breath analysis. Clin. Chem..

[2] Zhang Q., Wang P., Li J., Gao X. (2000). Diagnosis of diabetes by image detection of breath using gas‐sensitive laps. Biosens. Bioelectron..

[3] Tjoa S., Fennessey P. (1991). The identification of trimethylamine excess in man: Quantitative analysis and biochemical origins. Anal. Biochem..

[4] Moorhead K., Lee D., Chase J., Moot A., Ledingham K., Scotter J., Allardyce R., Senthilmohan S., Endre Z. (2008). Classifying algorithms for SIFT‐MS technology and medical diagnosis. Comput. Methods Progr. Biomed..

[5] Mayes P., Murray R., Granner D., Rodwell V. (2000). Harper's Biochemistry.

[6] Deng C., Zhang J., Yu X., Zhang W., Zhang X. (2004). Determination of acetone in human breath by gas chromatography-mass spectrometry and solid-phase microextraction with on-fiber derivatization. J. Chromatogr. B.

[7] Wang C., Weng Y., Chou T. (2007). Acetone sensor using lead foil as working electrode. Sens. Actuators B Chem..

[8] Penza M., Cassano G., Sergi A., Sterzo C.L., Russo M. (2001). SAW chemical sensing using poly—ynes and organometallic polymer films. Sens. Actuators B Chem..

[9] Ying Z., Jiang Y., Du X., Xie G., Yu J., Tai H. (2008). Polymer coated sensor array based on quartz crystal microbalance for chemical agent analysis. Eur. Polym. J..

[10] Huang H., Zhou J., Chen S., Zeng L., Huang Y. (2004). A highly sensitive QCM sensor coated with Ag^+^-ZSM-5 film for medical diagnosis. Sens. Actuators B Chem..

[11] Young D.J., Pehlivanoglu I.E., Zorman C.A. (2009). Silicon carbide MEMS-resonator-based oscillator. J. Micromech. Microeng..

[12] Kwok W., Bow Y., Chan W., Poon M., Wan P., Wong H. Study of Porous Silicon Gas Sensor.

[13] Li J., Lu Y., Ye Q., Cinke M., Han J., Meyyappan M. (2003). Carbon nanotube sensors for gas and organic vapor detection. Nano Lett..

[14] Chakraborty S., Banerjee D., Ray I., Sen A. (2008). Detection of biomarker in breath: A step towards noninvasive diabetes monitoring. Curr. Sci..

[15] Vomiero A., Bianchi S., Comini E., Faglia G., Ferroni M., Sberveglieri G. (2007). Controlled growth and sensing properties of In_2_O_3_ nanowires. Cryst. Growth Design.

[16] Wang L., Teleki A., Pratsinis S., Gouma P. (2008). Ferroelectric WO_3_ nanoparticles for acetone selective detection. Chem. Mater..

[17] Sahay P. (2005). Zinc oxide thin film gas sensor for detection of acetone. J. Mater. Sci..

[18] Lin Y.-S., Lin K.-H., Chang Y.-M., Yeh J.A. (2012). Epitaxy of m-plane GaN on nanoscale patterned c-plane sapphire substrates. Surface Sci..

[19] Wu C., Shen C., Lin H., Lee H., Gwo S. (2005). Direct evidence of 8: 9 commensurate heterojunction formed between InN and AlN on c plane. Appl. Phys. Lett..

[20] Lu Y.-S., Huang C.-C., Yeh J.A., Chen C.-F., Gwo S. (2007). InN-based anion selective sensors in aqueous solutions. Appl. Phys. Lett..

[21] Chang Y.-H., Lu Y.-S., Hong Y.-L., Kuo C.-T., Gwo S., Yeh J.A. (2010). Effects of (NH_4_)_2_S_x_ treatment on indium nitride surfaces. J. Appl. Phys..

[22] Lu Y.-S., Ho C.-L., Yeh J.A., Lin H.-W., Gwo S. (2008). Anion detection using ultrathin InN ion selective field effect transistors. Appl. Phys. Lett..

[23] Voss L., Gila B.P., Pearton S.J., Wang H.T., Ren F. (2005). Characterization of bulk GaN rectifiers for hydrogen gas sensing. J. Vac. Sci. Technol. B.

[24] Hsiao F.L., Lee C. (2010). Computational study of photonic crystals nano-ring resonator for biochemical sensing. IEEE Sens. J..

[25] Chang Y.-H., Chang K.-K., Gwo S., Yeh J.A. (2010). Highly sensitive hydrogen detection using a Pt-catalyzed InN epilayer. Appl. Phys. Express.

[26] Kryliouk O., Park H.J., Wang H.T., Kang B.S., Anderson T.J., Ren F., Pearton S.J. (2005). Pt-coated InN nanorods for selective detection of hydrogen at room temperature. J. Vac. Sci. Technol. B.

[27] Chang Y.-H., Chang K.-K., Gwo S., Yeh J.A. (2011). Highly sensitive pH sensing using an indium nitride ion-sensitive field-effect transistor. IEEE Sens. J..

[28] Lu H., Schaff W.J., Eastman L.F. (2004). Surface chemical modification of InN for sensor applications. J. Appl. Phys..

[29] Lin Y.-S., Yeh J.A. (2011). GaN-based light-emitting diodes grown on nanoscale patterned sapphire substrates with void-embedded cortex-like nanostructures. Appl. Phys. Express.

[30] Kakati N., Jee S.H., Kim S.H., Oh J.Y., Young S.Y. (2010). Thickness dependency of sol-gel derived ZnO thin films on gas sensing behaviors. Thin Solid Films.

[31] Ahn H., Wang Y., Jee S.H., Park M., Yoon Y.S., Kim D.-J. (2011). Enhanced UV activation of electrochemically doped Ni in ZnO nanorods for room temperature acetone sensing. Chem. Phys. Lett..

[32] Murade P.A., Sangawar V.S., Chaudhari G.N., Kapse V.D., Bajpeyee A.U. (2011). Acetone gas-sensing performance of Sr-doped nanostructured LaFeO3 semiconductor prepared by citrate solegel route. Curr. Appl. Phys..

[33] Teleki A., Pratsinis S.E., Kalyanasundaram K., Gouma P.I. (2006). Sensing of organic vapors by flame-made TiO_2_ nanoparticles. Sens. Actuators B Chem..

[34] Bhuiyan A.G., Hashimoto A., Yamamoto A. (2003). Indium nitride (InN): A review on growth, characterization, and properties. J. Appl. Phys..

[35] Lu H., Schaff W.J., Eastman L.F., Stutz C.E. (2003). Surface charge accumulation of InN films grown by molecular-beam epitaxy. Appl. Phys. Lett..

[36] Dürr M., Höfer U. (2006). Dissociative adsorption of molecular hydrogen on silicon surfaces. Surf. Sci. Rep..

[37] Lundström I., Shivaraman S., Svensson C., Lundkvist L. (1975). A hydrogen-sensitive Pd-gate MOS transistor. Appl. Phys. Lett..

[38] Deng L., Ding X., Zeng D., Tian S., Li H., Xie C. (2012). Visible-light activate mesoporous WO_3_ sensors with enhanced formaldehyde-sensing property at room temperature. Sens. Actuators B.

[39] Gonga J., Chena Q., Lian M.-R., Liu N.-C., Stevenson R.G., Adami F. (2006). Micromachined nanocrystalline silver doped SnO_2_ H_2_S sensor. Sens. Actuators B Chem..

[40] Moon K.H., Hong H.K., LG ELECTRONICS INC. (2005). Gas sensor has dehumidifying part for removing moisture using layered thin film-type dehumidifying agents made of paper.

